# Metabolic Surgery for Type 2 Diabetes with BMI <35 kg/m^2^: An Endocrinologist’s Perspective

**DOI:** 10.1007/s11695-013-0907-1

**Published:** 2013-03-22

**Authors:** Harold E. Lebovitz

**Affiliations:** Department of Medicine, State University of New York Health Science Center at Brooklyn, Brooklyn, NY USA

**Keywords:** Metabolic surgery, Type 2 diabetes, Body mass Index <35 kg/m^2^, Remission of diabetes

## Abstract

Is bariatric surgery as primary therapy for type 2 diabetes mellitus (T2DM) with body mass index (BMI) <35 kg/m^2^ justified? Open-label studies have shown that bariatric surgery causes remission of diabetes in some patients with BMI <35 kg/m^2^. All such patients treated had substantial weight loss. Diabetes remission was less likely in patients with lower BMI than those with higher BMI, in patients with longer than shorter duration and in patients with lesser than greater insulin reserve. Relapse of diabetes increases with time after surgery and weight regain. Deficiencies of data are lack of randomized long-term studies comparing risk/benefit of bariatric surgery to contemporary intensive medical therapy. Current data do not justify bariatric surgery as primary therapy for T2DM with BMI <35 kg/m^2^.

## Introduction

The worldwide epidemic of type 2 diabetes is increasing at a rate far exceeding previous predictions. It is now projected that the number of persons with diabetes in 2030 will be 552 million [1]. Since diabetes is the cause of 40–55 % of end-stage renal disease, 50–60 % of coronary artery disease, a preponderance of visual loss in adults and the majority of non-traumatic lower extremity amputations, its impact on population health and health care costs are and will be increasingly enormous [2]. It is within this context that we are struggling to develop strategies to prevent diabetes and treatments to reduce its chronic complications.

The great increase in type 2 diabetes has been due to environmental changes, many of which have resulted in obesity associated with the metabolic syndrome and all of its consequences. The prevention and/or the treatment of obesity results in decreases in the development of type 2 diabetes and, where diabetes already exists, marked improvements in its metabolic abnormalities [3–5]. Unfortunately, lifestyle modification with or without pharmacologic therapy has been relatively ineffective as a therapeutic endeavor to treat obesity.

A consequence of the ineffective medical therapy for obesity has been the development of a number of surgical procedures to cause and maintain weight loss [6, 7]. These procedures have been termed bariatric surgery and have been quite effective in reducing morbidity and mortality in individuals with severe obesity [8–12]. As approximately 20 % of individuals with severe obesity have concomitant type 2 diabetes, it has been possible to show that bariatric surgery results in marked improvement of diabetes [10, 12].

More recently, many studies have indicated that bariatric surgery in patients with type 2 diabetes and type 2 (body mass index [BMI] 35 to <40 kg/m^2^) or type 3 obesity (BMI ≥40 kg/m^2^) causes remission or improvement of the diabetes [7, 8, 10, 12]. As a result of these findings, the obvious question to be asked is whether bariatric surgery should be considered as a primary treatment for diabetes in overweight and type 1 obese individuals with type 2 diabetes. Evidence for and against the use of bariatric surgical procedures to treat the ordinary individual with type 2 diabetes is the subject of this analysis.

## The Role of Obesity in Generating Metabolic Abnormalities of Type 2 Diabetes

Before we can address the role that obesity plays in the pathogenesis of the metabolic abnormalities of type 2 diabetes, we need to have a definition of obesity. The current classification of obesity is based on the BMI, which is defined as weight in kilograms divided by height in meters squared [13]. A BMI from 25.0 to 29.99 is defined as overweight and a BMI greater than 30.0 is defined as obese [13]. Obesity is further subdivided into type I (BMI 30.0 to 34.99 kg/m^2^), type II (BMI 35.0 to 39.99 kg/m^2^) and type III (BMI greater than 40.0 kg/m^2^)[13]. These criteria, however, fail to take into consideration the percentage of body fat or distribution of body fat and therefore are only moderately correlated with true body fatness.

The metabolic abnormalities associated with an increase in body fat (metabolic syndrome and insulin resistance syndrome) are correlated most strongly with hepatic triglyceride content and visceral adiposity mass rather than subcutaneous adiposity or total body fat [14, 15]. It is the insulin resistance and its associated abnormalities (dyslipidemia, increased blood pressure, endothelial dysfunction and pro-coagulant state) that drive the development and progression of type 2 diabetes in individuals with genetically predisposed beta cell abnormalities [16]. In a study of 538 obese individuals with fasting plasma glucose (FPG) ≤120 mg/dl and BP <160/90 mm of Hg, euglycemic hyperinsulinemic clamps showed that insulin resistance was present in only 34 % of individuals with BMI <35 kg/m^2^, but in 60 % of those with BMI >35 kg/m^2^ [17].

Overweight and obese individuals therefore can be divided into those with and without metabolic disorders. The prevalence of the metabolic syndrome in individuals with type 2 diabetes is approximately 80 %.

## Mechanisms by Which Bariatric Surgery Improves Hyperglycemic and Cardiovascular risk Factors in Patients with the Metabolic Syndrome and Type 2 Diabetes

The current clinically accepted bariatric surgical procedures are laparoscopic gastric banding (LGB), Roux-en-Y gastric bypass (RYGB), sleeve gastrectomy and bilio-pancreatic diversion with a duodenal switch (BPD/DS). The mechanisms by which these procedures exert their effects are food restriction, malabsorption and alterations in upper and lower gastrointestinal hormonal and metabolic factors [7, 18–20]. Gastric banding is a restrictive procedure and decreases hyperglycemia proportional to the magnitude and time course of weight loss [18]. Sleeve gastrectomy appears to have both restrictive effects through food restriction and weight loss and additionally alterations in lower gastrointestinal hormone and metabolic factors. RYGB which is primarily a diversionary procedure improves hyperglycemia within several weeks of the surgery and before significant weight loss has occurred suggesting that non-weight loss factors, such as alterations in upper and lower gastrointestinal metabolic factors, are responsible for a significant component of the observed clinical benefits [7, 18–20]. BPD/DS benefits are due to a combination of diversionary effects plus a significant malabsorption component [7].

A major issue in assessing the mechanisms responsible for the improvements in diabetes in diversionary surgical procedures is the difficulty in quantifying the effects of food restriction itself from the other non-weight loss components of the various bariatric surgical procedures. Short-term experiments in patients with type 2 diabetes have demonstrated that caloric restriction, independent of weight loss, improves glycemic control [21]. A recent study in obese patients with type 2 diabetes put on a 600-kcal diet for 8 weeks showed rapid improvement in FPG and HbA1c, which were observed after 1 week (Table 1) despite the small changes in body weight and BMI [22]. The most dramatic changes after 1 week of caloric restriction were the decrease in liver triglycerides and in fat mass (Table 1) [22]. After 8 weeks, FPG and HbA1C were normalized [22]. Body weight and BMI were decreased 14.8 % and 14.6 %, respectively, but again the most dramatic effects of the caloric restriction were a 77 % decrease in hepatic triglycerides and a 33 % decrease in body fat (Table 1) [22].

**Table 1:** Metabolic effects of an 8-week 600 kcal/day in 11 patients with type 2 diabetes

Parameter	Baseline	Week 1	Week 4	Week 8
Weight (% baseline)	100	96.1	90.7	85.2
BMI (% baseline)	100	96.1	90.8	85.4
Fat Mass (% baseline)	100	93.8	81.3	67.4
Hepatic Triglyceride (% baseline)	100	70		23
Fasting plasma glucose (mmol/l)	9.2	5.9	5.7	5.7
HbA1C (%)	7.4	7.1	6.5	6.0

Baseline characteristics: weight 103.7 ± 4.5 kg, BMI 33.6 ± 1.2 kg/m^2^, fat mass 39.0 ± 3.5 kg, hepatic triglyceride 12.8 %. Data are from Lim et al. [22]

It is apparent that caloric restriction after complex surgical procedures can account for at least part of the early benefits in improving hyperglycemia in patients with type 2 diabetes. The role of changes in ghrelin, GLP-1 and PYY secretion following diversionary surgical procedures need to be defined by physiologic experiments rather than just by plasma measurements.

## Is BMI the Best Parameter for Selection of Patients for Metabolic Surgery for the Treatment of Type 2 Diabetes?

The metabolic abnormalities of type 2 diabetes are ameliorated either by an increase in insulin availability or a decrease in insulin resistance. Weight loss in obese individuals with insulin resistance will improve insulin sensitivity and in those with diabetes improves hyperglycemia [4, 22]. Improving hyperglycemia decreases glucose toxicity, which improves insulin secretory function. Insulin resistance in overweight and obese individuals is caused by the disposition of excess quantities of lipids, lipid metabolites and adipokines into non-adipose tissue cells such as muscle, liver and endothelial cells [23]. These excess lipid deposits block the intracellular action of insulin and result in insulin resistance [23]. Recent studies indicate that insulin resistance is secondary to an increase in hepatic triglyceride content as well as an increase in visceral adiposity [14, 15, 23]. Decreasing hepatic steatosis and reducing visceral adipose tissue mass decreases insulin resistance. While BMI partially reflects adipose tissue mass, it is also determined by non-adipose tissue factors and is not a good measure of either hepatic triglyceride content or visceral adipose tissue mass. BMI therefore, while an easily obtainable and cheap measurement is nonetheless a poor index of metabolically significant obesity [24].

The use of BMI to determine the potential benefits of metabolic surgery for resolution or improvement in diabetes management therefore should not be considered a stringent criterion as it is not a measure of metabolically significant abnormalities associated with obesity [14, 15, 24].

## How Important Is Weight Loss in the Resolution of Diabetes by Bariatric Surgery in Individuals with BMI <35 kg/m^2^?

Relatively few studies have examined the effects of bariatric surgery in patients with type 2 diabetes and BMI <35 kg/m^2^ [25]. Table 2 lists data from four recent studies. Lee and colleagues [26] examined the data from their large series of laparoscopic mini-gastric bypass surgery reported the results of those with baseline BMI <35 kg/m^2^ and those with BMI >35 kg/m^2^. The percent weight loss was almost as great in those with baseline BMI <35 kg/m^2^ as it was in those with >35 kg/m^2^. Cohen and colleague’s [27] patients with baseline BMI <35 kg/m^2^ lost 35–40 % of their body weight in 1 year [27]. Similarly, Schauer et al. [28] though not giving specific details for their patients with BMI <35 kg/m^2^ reported weight losses of approximately 25 % in their surgically treated patients. Lee et al. [29] in a multicenter study of 200 patients with diabetes and BMI <35 kg/m^2^ reported on a 1-year follow-up of 87 patients, most of whom had gastric bypass surgery, that the mean body weight loss was 19.4 %.

**Table 2:** Weight loss following gastric bypass surgery in patients with BMI <35 kg/m^2^ and 1 year follow-up

Author	Number of patients with BMI <35 kg/m^2^	Mean BMI (kg/m^2^)	Mean weight (kg)	Weight loss 1 year		Weight loss 5 years
				(kg)	% body wt	
Lee et al. [26]	44 (21.9 %)	31.7		24	30 %	Approximately 36 %
Schauer et al. [28]						
Gastric bypass	14 (28 %)	37.0	106.7	29.4	27.6 %	
Sleeve gastrectomy	18 (36 %)	36.1	100.8	25.1	24.5 %	
Cohen et al. [27]	66	32.7			36 %	
					40 %	
Lee et al. [29]	87	28.5			19.4 %	
Gastric bypass	86 %			20.3		
Restrictive procedure	14 %			15.4		

It is therefore difficult to make a major distinction between the effects of gastric bypass surgery and sleeve gastrectomy on the magnitude of body weight reduction between patients with type 2 diabetes with BMI <35 and >35 kg/m^2^ as both groups have a striking decrease in body weight. The magnitude of weight loss has to be a major factor in the improvement of metabolic abnormalities in obese patients with type 2 diabetes irrespective of whether their baseline BMI is <35 or >35 kg/m^2^. However, the magnitude of improvement in diabetes does appear to be less in those with the lower BMI as compared to those with the higher BMI. The same appears to be true with the recurrence rate of diabetes after remission, being higher in the lower baseline BMI population than in the higher baseline BMI population.

## Results of Bariatric Surgery in Patients with Type 2 Diabetes: BMI <35 kg/m^2^ as Compared to >35 kg/m^2^

The glycemic benefits of metabolic surgery in patients with type 2 diabetes and BMI <35 kg/m^2^ have been reported in several recent publications. The results of early postoperative outcomes from the ASMBS Bariatric Surgery Center of Excellence Program have been reported on 235 patients operated upon from July 2007 to June 2009 with BMI ≥30 but <35 kg/m^2^ with diabetes requiring treatment with medications [30]. Comparisons were carried out between 109 who had adjustable gastric banding and 109 patients who had gastric bypass surgery. Laparoscopic access was used in 92 % of the patients. The diabetes pre-operatively had been treated with oral agents alone (gastric bypass 53.3 %; adjustable gastric banding 66.1 %), or insulin alone or with oral agents (gastric bypass 43.1 %; adjustable gastric banding 33.1 %). Follow-up data were available for 6–12 months in a little more than 50 % of the patients. The BMI decreased from 33.1 to 27.2 (14.5 %) at 6 months in the gastric bypass group and from 33.9 to 31.0 (8.6 %) in the adjustable gastric banding group. Percent excess body weight decreased from 57 % to 26.8 % after gastric bypass and 45.5 % after adjustable gastric banding. The effect on diabetes was not measured by changes in HbA1C or FPG but rather by the change in diabetic treatments. After 6 to 12 months, 50 % of gastric bypass and 31.8 % of adjustable gastric band patients were no longer taking diabetes medications. Among patients initially taking insulin plus oral medications, 11.1 % and 50 % had no need for diabetes medications 6 months after adjustable gastric banding and gastric bypass, respectively. The number of significant postoperative complications through 90 days reached 20 following gastric bypass and three following adjustable gastric banding.

**Fig. 1:**
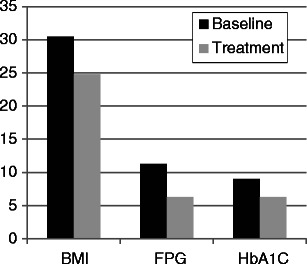
The results of metabolic surgery in patients with type 2 diabetes and BMI <35 kg/m^2^. The data are the result of meta-analyses from 14 studies for the change in BMI, 12 studies for the change in fasting plasma glucose (*FPG*) and ten studies for the change in HbA1C. Data are derived from Shimizu et al. [31]

Shimizu et al. [31] have reviewed and summarized 18 published studies involving 477 patients with type 2 diabetes and BMI <35 kg/m^2^ that were treated surgically for type 2 diabetes. Of the 18 studies, seven were done in Brazil, four in Italy, four in Taiwan, one in Chile, one in the US and one in India. Surgical procedures included RYGB in six, duodenal–jejunal bypass in four, biliopancreatic diversion (BPD) in three, mini gastric bypass in two, ileal interposition with sleeve or diverted sleeve gastrectomy in two, sleeve gastrectomy in one and stomach and pylorus-preserving BPD in 1. Thirty percent of the patients had been treated with insulin prior to the surgical procedure. The follow-up period ranged from 6 to 216 months. The mean changes in BMI, FPG and HbA1C for the studies in which they were reported are shown in Fig. 1. Note that the mean BMI decreased from 30.4 ± 0.98 to 24.8 ± 0.33 kg/m^2^. Remission of diabetes as defined by FPG <126 mg/dl and HbA1C <6.5 % without the use of antidiabetic medications [32] was reported in 64.7 % of patients. When patients were stratified by duration of diabetes at the time of surgery, insulin use was 18.2 % in those with diabetes duration ≤8 years and 45.9 % in those >8 years and the remission rates after surgery were 66 % in those with the shorter duration and 52.9 % in those with the longer duration of diabetes. Postoperative complications occurred in 10.3 % of the patients with no reported mortalities. The risk of excessive weight loss after surgery was 2.7 %.

**Fig. 2:**
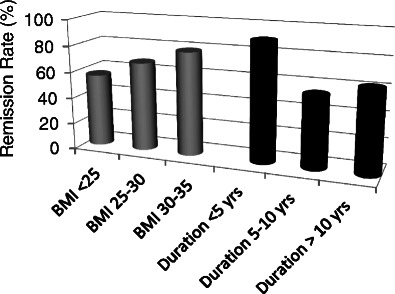
The relationship between baseline BMI and baseline duration of known diabetes with the percent of patients with remission of their diabetes 1 year after metabolic surgery. The data are derived from 87 of 200 patients who had achieved a 1-year follow-up in a multi-institutional international Asian study. Data are derived from Lee et al. [29]

Lee and colleagues have reported the results of a multi-institutional study of metabolic surgery in 200 Asian patients with type 2 diabetes and BMI <35 kg/m^2^. One hundred seventy two patients had a gastric bypass, 24 sleeve gastrectomy, and four adjustable gastric banding. Mean baseline HbA1C was 9.3 ± 1.9 % and the end point, which was remission of diabetes, was defined as FPG <110 mg/dl and HbA1C <6.0 % in the absence of any diabetic medications. One-year data were available for 87 patients [29]. BMI decreased from 28.5 ± 3.0 to 23.4 ± 2.3 kg/m^2^, body weight loss was 19.4 %, and HbA1C decreased to 6.3 ± 0.5 %. Remission of diabetes was achieved in 72.4 % of the patients. The remission rate was higher in those with diabetes duration <5 years (90.3 %) compared to those with duration >5 years (57.1 %); those with BMI >30 kg/m^2^ (78.7 %) than those <30 kg/m^2^ (62.5 %) and in those following gastric bypass than in those following restrictive-type procedures (Fig. 2). Following metabolic surgery, the patients had significant improvement in waist circumference, systolic and diastolic blood pressure, total and LDL-cholesterol, triglycerides and HOMA-IR.

The longest and most complete follow-up on patients with type 2 diabetes with BMI <35 kg/m^2^ who have been treated by metabolic surgery was recently published by Cohen and associates [27]. They did a gastric bypass in 66 patients with long-duration (12.5 ± 7.4 years) of type 2 diabetes poorly controlled on medical therapy (mean HbA1C 9.7 ± 1.1.5 %). The follow-up rate which extended up to 6 years was 100 %. Durable diabetes remission (HbA1C <6.5 % without diabetes medications) has occurred in 88 % of the patients with improvement in glycemia in an additional 11 %. The mean HbA1C after surgery was 5.9 ± 0.1 %. The majority of patients discontinued diabetes medications. The initial mean BMI was 32.7 kg/m^2^. The mean weight loss at last follow-up was 37 % in those whose diabetes improved and 40 % of those whose diabetes went into remission. Mean waist circumference decreased from 123 to 88 cm in those with type 2 diabetes resolved and 85 cm in those with diabetes improved. Associated metabolic improvements following surgery were noted in systolic and diastolic blood pressure, total, LDL and HDL cholesterol and triglycerides.

The data indicate that gastric bypass surgery is effective as a treatment for type 2 diabetes with BMI 30 to 34.9 kg/m^2^. The data which are available suggest that the benefits in diabetes resolution and improvement are not as great in those with BMI <35 as in those with BMI >35 kg/m^2^. In all BMI categories, shorter duration of diabetes, less severe baseline hyperglycemia and oral agent therapy are predictors of better resolution of diabetes following metabolic surgery.

## Recurrence of Diabetes After Metabolic Surgery Induced Remission

Long-term follow-up of patients with type 2 diabetes who have had diabetes remission or significant improvement show that there is a recurrence rate that increases with length of time after the surgery (Table 3). In the Swedish Obesity Study (SOS), the remission rate for diabetes after bariatric surgery was 72 % after 2 years compared to a control population where it was 21 %. However, after 10 and 15 years of follow-up, the diabetes remission rate had fallen to 36 % and 30 %, respectively, for the surgically treated group and was 13 % and 6 %, respectively, for the control group [8]. The diabetic population in the SOS study had a mean initial BMI 42.1 kg/m^2^. Diabetes remission following surgery correlated with a shorter duration of diagnosed diabetes at baseline and a larger weight loss at 2 years. Relapse of diabetes remission was associated with longer diabetes duration at baseline, higher baseline body weight, higher baseline plasma glucose, smaller weight loss at 2 years and a smaller reduction of plasma insulin at 2 years [8].

**Table 3:** Re-emergence of diabetes after resolution with bariatric surgery during long-term follow-up

Author	Surgical procedure	Number of patients with diabetes	Number (%) with remission of diabetes	Number (%) with relapse of diabetes	Time at relapse (years)
Sjostrom et al. [8]	Vertical banded	345	229 (72 %)	0 %	2 years
	gastroplasty (71 %)			50 %	10 years
	Gastric banding (24 %)			58.3 %	15 years
	Gastric bypass (5 %)				
Kim and Richards [36]	Gastric bypass	219	156 (71 %)	11 (7.1 %)	2 to 5 years
DiGiorgi et al. [34]	Roux-en-Y-gastric bypass	42	27 (64 %)	10 (24 %)	≥3 years
Chikunguwo et al. [33]	Roux-en-Y gastric bypass	177	157 (89 %)	68 (43 %)	Within 5 years
Ramos et al. [35]	Not specified	72	66 (91.6 %)	14 (21.2 %)	5 < 2 years
					3 at year 3
					3 at year 4
					3 at year 5

In Chikunguwo et al.'s series, durable remissions were most likely to occur in men, early in the time course of diabetes and in those controlling their diabetes with diet and/or oral hypoglycemic agents [33]. DiGiorgi and associates [34] noted that lower preoperative BMI, a greater weight loss failure rate, regain of a greater percentage of the lost weight, and a higher postoperative glucose level predicted recurrence of the diabetes. Patients who required insulin or oral medications before metabolic surgery were more likely to have improvement in their diabetes rather than remission of their diabetes. In Ramos et al.’s study [35], preoperative diabetes duration predicted late recurrence. In the subset of patients with diabetes duration of 5 years or longer, the odds ratio for recurrence was 3.8.

The recurrence data [8, 33–36] can be summarized as showing that long duration of diabetes, more severe hyperglycemia and more intense medical treatment preoperatively predict a higher rate of long term recurrence of diabetes in those who initially have a remission of their diabetes. These data suggest that bariatric surgery slows but does not prevent the progressive deterioration of beta bell function in patients with type 2 diabetes.

## Surgical and Metabolic Complications Following Metabolic Surgery

Equally as important as the remission and improvement rate of diabetes following bariatric surgery are the rates and severity of complications from the surgical procedures and the long term outcomes of these procedures. Relatively little data are available on the short and long-term complications of bariatric surgical procedures because of the paucity of long-term follow-up studies and the inordinately large dropout rates of most of the studies that are reported. While the long-term outcome data of bariatric surgery in the severely obese is inadequate, it is virtually non-existent in individuals with type 2 diabetes with BMI <35 kg/m^2^.

A recent review of early outcomes of bariatric surgery from the Bariatric Outcomes Longitudinal Database [37] compared the results in patients with the metabolic syndrome (23,106) to those without the metabolic syndrome (163,470). Gastric bypass had been done in 62 % of the patients and gastric banding in 32 %. Mean baseline BMI was 46.9 kg/m^2^. Compared to patients without the metabolic syndrome, patients with the metabolic syndrome had an increase in serious complications (2.4 to 1.0 %), readmissions to the hospital (6.2 to 4.7 %) and mortality (0.3 to 0.1 %) within 90 days of operation. The 12 months remission rate for diabetes in the metabolic syndrome patients who had had gastric bypass surgery was 62 %

A study comparing complications of gastric bypass to gastric banding with a 92.3 % follow-up at 6 years in 442 patients matched for sex, age and BMI showed that RYGB had a higher early (17.2 % vs. 5.4 %) but lower late complication rate than gastric banding (LB) [38]. Total complications following RYGB occurred in 42 patients (19 %) and included anastomotic strictures, marginal ulcers, small bowel obstruction secondary to adhesions or internal hernias and symptomatic small bowel or incisional hernias [38]. Re-operation was necessary in 12.7 % of patients. In the gastric band patients, total complications were 92 (41.6 %) and included port/catheter leak, band leak, port or band infection, band erosion, pouch or esophageal dilatation, gastric esophageal reflux, and food intolerance [38]. Re-operation was necessary in 26.7 % of the patients and band removal was necessary in 47 (21.3 %) of the patients. RYGB had a greater 6-year weight loss and success rate than gastric banding. Other studies have shown similar complication rates for the two procedures in severely obese patients [39–43].

Table 4 lists the complications of gastric bypass surgery in three recently published series with somewhat lower mean BMI and a 1-year follow-up [26, 28, 44]. The results parallel those of earlier studies with more severely obese individuals.

**Table 4:** Reported short-term (1 year) complications in several recently published clinical trials of bariatric surgery in patients with type 2 diabetes

Complication	Gastric bypass		
	DeMaria et al. 90 days after operation 109 patients [30]	Schauer et al. 1 year 50 patients [28]	Mingrone et al. 9–18 months after operation 19 patients [44]
Anastomotic leak	1		
Anastomotic stricture	4		
Anastomotic ulceration		4	
Anemia		6	2
Hemoglobin decrease >5 g/dl		1	
Cholelithiasis		1	
Common bile duct obstruction	1		
Gastrointestinal bleeding	1		
Hypokalemia		2	
Internal hernia	1	1	
Intestinal obstruction			1
Intravenous treatment for dehydration		4	
Ketoacidosis		1	
Nausea and vomiting	4		
Nutritional disturbances	1		
Pneumonia	2	2	
Atelectasis	1		
Re-operation		3	
Requiring hospitalization		11	
Surgical wound infection	1	1	
Transient renal insufficiency		1	
Transfusion		1	
Vitamin K deficiency	1		

Another aspect to the short- and long-term complications of bariatric surgery are the nutritional disturbances which can and frequently are the consequences of alterations in gastrointestinal physiology which occur after the various surgical procedures. These have been reviewed extensively in other publications [45–49]. They can lead to acute syndromes such as Wernicke’s syndrome and protein malnutrition, and more chronic problems such as iron deficiency with anemia, vitamin D and calcium deficiency with bone disease, vitamin B_12_ and folate deficiencies, vitamin A deficiency, as well zinc, magnesium selenium, and copper deficiencies.

## Conclusions

After having evaluated the available evidence, what conclusions can we reach concerning the role of metabolic surgery as a primary treatment for patients with BMI <35 kg/m^2^ and type 2 diabetes. Bariatric surgery resolves or improves hyperglycemia and the metabolic syndrome in a majority of type 2 diabetic patients with BMI 30 to 35 kg/m^2^. The beneficial effects on diabetes are most likely to occur in those with diabetes duration <5 years who still have significant beta cell functional reserve as demonstrated by some responsiveness to dietary and oral agent therapies. The lower the BMI in the type I obesity and overweight range, the less responsive the diabetes will be to bariatric surgery. Though non-weight loss factors play a role in the improvement seen in the diabetes in patients with BMI <35 kg/m^2^, all studies with the traditional bariatric surgery procedures have shown remarkable weight loss — frequently restoring the BMIs of patients to within the normal weight range. Since significant weight loss by medical and/or dietary therapy in this population can achieve the same short-term results as bariatric surgery [21, 22, 50], it might be reasonable to speculate that the major difference between bariatric surgery and dietary management is the lack of sustainable effect in dietary management versus the sustainability of weight loss by gastric bypass or other bariatric surgical procedures.

Evidence that bariatric surgery does not stop — but rather, slows down — the progression of the beta cell decline in patients with type 2 diabetes are the data that bariatric surgery induced remission of diabetes lessens with time and that relapses of the diabetes are being reported with increasing frequency as more long-term follow-up data are being reported (Table 3).

The biggest drawback to recommending bariatric (metabolic) surgery for treating patients with diabetes and BMI <35 kg/m^2^ are the lack of randomized, controlled clinical trials comparing contemporary intensive lifestyle and pharmacologic treatment to bariatric surgical treatment. Several recent papers have discussed the issues of medical therapy for patients with type 2 diabetes [51, 52]. Large clinical trials have been able to maintain HbA1C at 6.5–6.9 % for periods as long as 3 to 7 years. With newer agents for weight loss and glycemic control about to enter the market place, metabolic and weight control should improve. Additionally, several very important clinically meaningful questions need to be answered as we evaluate the benefits of medical versus metabolic surgery treatments. Are HbA1C levels <6.5 % necessary to reduce diabetic complications or is <7 % adequate? How much weight loss in patients with BMI 30 to 35 kg/m^2^ is necessary to achieve the metabolic benefits of weight loss? Could it be as little as 5–10 % of body weight?

An even more vexing question is whether the benefits of bariatric surgery in patients with type 2 diabetes and BMI 27–35 kg/m^2^ are sufficient to justify the side effects of the procedures [51, 53]. An operative mortality of 0.2–0.3 % means that for every 1,000 patients with type 2 diabetes and BMI <35 kg/m^2^, there will be two to three deaths. This is unacceptable for a disease that can be effectively treated medically. As shown in Table 4, even in the best surgical centers, complications following RYGB surgery are very frequent and significant. If randomized, controlled studies of bariatric surgery versus the best contemporary medical management show that long-term clinical outcomes are better and that the benefit/risk ratio is greater with bariatric surgery than the best contemporary medical treatments, we will have a strong argument for metabolic surgery as a primary therapy for ordinary type 2 diabetes.

Until we have such data, which will take many years to obtain, what should be our current position regarding metabolic surgery as a treatment for type 2 diabetes in patients with BMI <35 kg/m^2^? Since diabetes regulation and cardiovascular risk factor reductions respond well to bariatric surgery in patients with type 2 diabetes with BMI ≥27 and <35 kg/m^2^, it should be considered as an option in those patients that cannot be adequately controlled on intensive medical therapy. The more obese the type 2 diabetic patient is, the more likely the potential benefit that can be obtained with metabolic surgery. The less overweight the patient, the less likely that there will be a meaningful benefit. In any event, a thorough discussion with the patient about the risks as well as the potential benefits needs to be discussed. Metabolic surgery is not a benign procedure and should only be undertaken if adequate intensive medical therapy is failing.
